# Extra Supplement.—The Nursing Mirror

**Published:** 1894-02-03

**Authors:** 


### "The Hospital" Nursing Mirror.

Being the Extra Nursing Supplement of "The Hospital" Newspaper.

[Contributions for this Supplement should be addressed to the Editor, The Hospital, 428, Strand, London, W.C., and should have the word "Nursing" plainly written in left-hand top corner of the envelope.]

### News from the Nursing World.

### A ROYAL VISIT TO PORTSMOUTH.

Princess Louise and Princess Henry of Battenberg were present on 30th January at the annual meeting held in Portsmouth Town Hall of the District Nursing Association, which is affiliated with the Queen's Jubilee Nursing Institute. Their Royal Highnesses lunched at the Admiralty House before the meeting, which was a largely-attended and most successful one. A concert was given the same evening in aid of the Royal Portsmouth, Portsea, and Gosport Hospital, under the patronage of the Duke and Duchess of York, the Queen and the Prince of Wales having bought a number of tickets.

### THE CHILDREN'S HOSPITAL.

The Duke of York has promised to preside at the dinner arranged to take place in aid of the funds of the Hospital for Sick Children, Great Ormond Street, on May 5th. The subscriptions following on this function (at the Hotel Metropole) will doubtless largely benefit the finances of this well-known hospital. Since the days when two picturesque old houses secured shelter and medical aid to many little sufferers, the Children's Hospital has appealed irresistibly to public sympathy. Changes have taken place and progress has been made in nursing since then, and the pretty garden with its fine old trees have disappeared, as have the nurses of that period. The night nurses used to sleep in the basement, which possibly did not furnish an altogether ideal dormitory. The nurses are now promoted to a higher level, and they are fairly well lodged, but the probationers' quarters are by no means up to date. Possibly before the day fixed by the Royal President for the dinner and its appeal the authorities will see their way to some modification of present arrangements. For eight or nine adults to occupy one room with inadequate partitioning of woollen curtains does not meet the views of subscribers or of workers. Yet the spacious halls, numerous corridors, staircases, and a large empty ward prove that no lack of space cramps the Children's Hospital, which ought to take first rank in accommodating its staff as well as its patients.

### ST. THOMAS'S HOSPITAL NURSES' CONCERTS.

On January 24th and 25th two excellent concerts were given in the fine hall at St. Thomas's Hospital to the nurses and their friends. The room was crowded by a most appreciative audience on each occasion, and the music was unusually good, even for a hospital, although these institutions always seem to be fortunate in possessing friends of exceptional musical ability, and great credit is due to the committee of doctors and students, whose admirable management and kindly consideration for their guests made the entertainments such unqualified successes.

### NURSES CO-OPERATION.

The third annual meeting of the Nurses Co-operation, 8, New Cavendish Street, will be held at 20, Hanover Square, on Friday, February 2nd, at half-past four. The chair will be taken by the Hon. Mr. Justice Kekewich, and speeches will be made by Sir William Broadbent, Bart., Mr. Thomas Bryant, Dr. Goodhart, Mr. Henry Kimber, and others.

### THE MIDWIVES' INSTITUTE.

The Midwives' Institute is just entering on its thirteenth year of existence, and the general meeting on January 27th, at 12, Buckingham Street, Strand, was very well attended. The chair was taken by Miss Hubbard (one of the original founders), and the report of its work is certainly most satisfactory.

### SUNDAY DINNERS.

Who has not seen the comely joint resting on a goodly supply of potatoes on its way to the baker's oven? Or the pie of fine dimensions, which causes the hungry man who encounters it as it emerges smoking from the shop door to remember that dinner time approaches? These Sunday dinners are altogether commendable, but they are not "the common lot of all.' There are a great many "Sunday dinners" prepared and eaten which do not court the inspection boldly invited by "the family leg of mutton" or the noble "steak and kidney pie." It is on behalf of these humbler meals, these ill-cooked and unappetising compositions that Mrs. John Elder, of Govan, established a housewifery school ten years ago. Such a school should certainly be possible of imitation for the benefit of an enormous class of women and girls. At Govan they are taught to cook food of their own providing in their own utensils. The cost of each dinner must be kept within a definite limitation of price. From half-past two to half-past eleven every Saturday these practical classes are held, and every woman and girl has time and opportunity to prepare a dinner for Sunday which will require only heating up to be ready for use. Variety and emulation are encouraged, and great is the competition in new dishes and nicely-laid meals. The plan, which has for so long been carried out successfully, is deserving of all praise, and must be included amongst the many notable schemes which aim at that highest kind of instruction which consists in teaching poor people to help themselves.

### WHEN CAN I SEE THE MATRON?

Naturally this is the regular question of the candidate for a vacancy in a training school, and to it she receives a variety of answers. At King's College Hospital two mornings a week are devoted by the matron to the important duty of interviewing applicants. At St. Thomas's an equally liberal amount of time is allotted to it. At the London Hospital stated times are specified and appointments made. Whilst at St. Bartholomew's one half hour a week is considered sufficient to settle the claims of the wouldbe nurses. In answer to the numerous applicants who ask us for information on the subject of training, we strongly advise that they should in all cases write to the matrons of several of the "schools," and then carefully and intelligently compare the rules and timetables of work given by each, before not after, inquiring, "When can I see the Matron?"

### PAUPERS STILL NEGLECTED.

The evils existing in Wolverhampton Workhouse were noticed in The Hospital of June 24th, 1893, under the heading "Neglected Paupers." Seven Guardians were then opposed to the reforms urgently advocated by Dr. Totherick, whose report revealed disastrous neglect of the sick poor. The need for trained nurses has been again advocated, and the opposition at the meeting came from five Guardians only. One of these said that if improvements were made in the nursing "everybody would go there." Let him dismiss that idea at once, for as long as he and his four supporters retain their official positions "everybody" will assuredly not flock into wards over which they have any power. A single nurse has had the charge of 240 aged and sick inmates, and the five wise men consider that these unfortunate creatures have been properly attended to. Happily Dr. Totherick holds juster views of the rights of the helpless poor. Neither he nor Mrs. Hatton, who gave valuable evidence, nor yet the majority of Guardians, see why the patients' food and stimulants have been withheld, even though they have been indulged in a supply of mixed medicines "outward" becoming "inward" applications in some instances. So the five wise men of Wolverhampton have before them the painful prospect of beholding the addition of four trained nurses to the staff. They must also submit to the sick and the dying receiving those common attentions which they seem to consider as "excessive nursing," and perhaps one of these wise men, who hold human life and suffering so cheap, may be induced to share the personal experiences of the inmates. Even under the new arrangements the proportion of nurses to patients leaves no margin for special nursing. At least one trained person must be on night duty, so that the others will have sixty or seventy cases each. Even a Guardian, if bed-ridden, might find a seventieth part of an overworked woman's attention hardly equals "excessive nursing." He might also discover that during her "time off" his own requirements by no means become conveniently suspended.

### AN ARISTOCRATIC DISEASE.

The ninth annual meeting of the Perth Sick Poor Nursing Society, which is affiliated with the Queen's Jubilee Institute, took place recently, and the report showed an excellent financial condition. One of the speakers said that influenza had been quite an aristocratic disease in that district, and the epidemic had not affected the poor. The nurses have nevertheless found plenty to do, and they have now arranged also to take over the district department hitherto managed by the infirmary. The authorities of the infirmary have promised a grant of £50 to the society, and they are in future to be absolved from providing either nursing or nourishment for the sick poor in their own homes. The Queen's Nurse will attend daily at the hospital and receive her instructions from the medical officers. It sounds a remarkably reasonable and well-organised scheme.

### TRAINED OR UNTRAINED?

The residents of Bangkok in Siam are expecting great things from the French Sisters who are undertaking the nursing of the hospital. No announcement is made as to the training of these ladies, although the press deals exhaustively with the subject of their religion. It is to be hoped that Bangkok has not copied Colombo in placing the charge of its hospital in the hands of women whose natural kindness and conventual training has not been supplemented by instruction and experience in the art of skilled nursing.

### A WELL-ORGANISED SCHEME.

The American Association of Superintendents of Training Schools for Nurses held their first annual meeting on the 11th inst. at the Academy of Medicine, New York. The next annual meeting will take place in Boston. The objects of this association are, speaking generally, to establish a common standard of instruction and length of training. The officers elected for the year (ladies well qualified by experience and knowledge to decide these important subjects) are: Miss Richards, Superintendent of the New England Training School, Boston, President; Miss Irene Sutliffe, of the New York Hospital, Vice-President; Miss M. S. Littlefield, Secretary; Miss Lucy L. Dronn, Treasurer, with a board of auditors and a council of six.

### SHORT ITEMS.

The two trained nurses at St. Austell's are doing excellent work amongst the sick poor.—The recommendation of the Nursing Committee concerning the staff at the Hampstead Workhouse Infirmary has been adopted by the Guardians, and a complete staff of trained nurses is to be organised, but unfortunately the present Lady Superintendent is untrained.—Dr. Lovell Drage has drawn the attention of the Local Government Board to the necessity for trained nurses at Hatfield Workhouse.—The sum of £1,500 has been left in trust by Dame Augusta Catherine Babier for the provision of a trained nurse for the sick of Uplyme; the nurse will be under the control of the Rector.—The annual report of the Coleraine Cottage Hospital is an excellent one. The services of the Queen's Nurse are greatly appreciated by the sick poor in the neighbourhood of Coleraine.—The annual report of the Radstock Sick Nursing Society makes cordial mention of the faithful and skilful service of Nurse Hoar.—At Leominster the work of the trained parish nurse is greatly appreciated, and the annual meeting of the Nursing Society was a most satisfactory one.—*Nursing Notes* for February contains an interesting Annus Medicus and other good papers, including excellent notes on cookery by Lucy Standon Price.—The annual ball at Swansea, held in aid of the hospital, was a great success this year.—Sir Henry Acland presented certificates on January 24th to the probationers at the Mile End Infirmary, who had passed the recent examination satisfactorily.—An extraordinary state of affairs seems to have been recently revealed at the Fever Hospital, Airdrie, and the Matron has been arrested on a charge of neglecting and abusing the patients.—The General Council of the Royal British Nurses' Association has conferred the Princess Helena Gold Medal of Merit for Nurses on Mrs. Bedford Fenwick "in recognition of her great services to the corporation in the advancement of nursing in this country, and of the signal success with which she filled the position of President of the British Nursing Section at the World's Columbian Exposition at Chicago."

### On Nursing the Nervous and Insane.

By T. DUNCAN GREENLEES, M.B.Edin., Medical Superintendent, Grahams Town Asylum, South Africa.

IV.—MASSAGE (*continued*).

No person should set up as a masseuse—for it is a trade or profession nowadays—without special training, for the procedure is not only complicated, but, to an inexperienced person, very exhausting.

Massage is the name applied to several different methods, to each of which brief reference will be made.

1. *Stroking* (*or effleurage*).—Here the points of the fingers or the palm of the hand start from a point on the distal side of the affected part, and, with slight pressure, are made to pass over the affected part to the proximal end. This motion is immediately followed by the other hand going through the same process, and so on in succession, one hand following the other at very short intervals.

2. *Massage à friction*, or rubbing, is a forcible pressure, with an accompanying motion of the hand, forwards. In this form the hand or fingers press upon the affected part and a circular motion is maintained, the pressure being kept up meanwhile.

In stroking, or *effleurage*, an endeavour is made to act upon the cutaneous surface, while in *friction* our object is to influence the deeper tissues, such as the muscles.

3. *Petrissage* (*or kneading*) consists in grasping a portion of tissue, generally an individual muscle, between the thumb and fingers, and, lifting it up as it were from its bed, exercising upon it a considerable amount of pressure. Both hands may be used in this process at the same time; the action is thereby rendered much more energetic, although it is more exhausting both to the operator and patient.

4. *Tapotement* (*or percussion*) is carried out either by the palm of the hand, the curved fingers, the closed fist, or by means of instruments specially constructed for this purpose, but whose action is similar to that of a hammer. When the hand is used, and the object is to percuss superficial nerves, then the action takes place from the wrist, in the same way as the chest is percussed, but when it is wished to reach deeper-seated tissues, then the wrist is fixed and the action takes place from the elbow, or even shoulder.

A masseuse should learn to use her hands equally well, for then the treatment is more continuous, and, for the most part, not so exhausting to the operator.

While it is quite possible for the massage treatment to be too severe, and even bruise marks result, there is the possibility of going to the other extreme and making it too mild or gentle; in this, as in all other things, there is a happy medium, which can only be learned by experience. The area of the body to be treated should be gone over thoroughly; one part should not be energetically massaged and the other only superficially so, perhaps on account of the operator feeling exhausted.

*Passive movements* of groups of muscles or of individual muscles may be ordered by the physician in certain forms of nerve disease or muscleatomy. In such cases "duplicated active movements," as they are called, are generally advised. Here the operator performs a movement, or a series of movements, with the patient's muscles, which the patient is enjoined to resist to the utmost of his strength. Thus, in the case of a partially paralysed arm, the arm is straightened out gradually, the patient all the while resisting the operator's attempts to flex it to the shoulder. The operator, during these efforts of the patient, should gradually give way to them. The arm is now flexed by the operator, who enjoins on the patient the necessity of resisting his efforts, and here again the operator permits his patient's efforts to overcome his own.

It is quite evident] that in many conditions, where the paralysis is local and not complete, such treatment, judiciously applied, and aided by the other methods previously referred to, should result in good to the palsied limb, and even, in time, recover its lost motor power.*

*c* BATHS.—In the treatment of nervous and mental diseases the bath, whether simple or medicated, is frequently made use of, and every institution admitting all classes of patients should possess a properly arranged series of baths. Further, it should form an essential part of every nurse's education to be thoroughly conversant with the different forms of baths in use, as well as the methods of applying them. This is a portion of the duties that is generally assigned without any medical aid whatever, as presumably nurses have already been instructed in the use of the bath in the treatment of many diseases such as fever; therefore, reference to them is only made so far as they apply in the treatment of nervous and mental cases.

In some spinal affections, such as myelitis and spinal irritation, the alternate application of heat and cold, or even iced water, to the spine is often beneficial. The wet pack is frequently used in delirium and mania; it should only be used, however, under the instructions of the physician, and the nurse should always remain with the patient during its application, for under its powerful influence shock to the nervous system and cardiac failure has been known to occur. After the removal of the pack the patient should be rolled up in warm blankets, the body having been previously rubbed down with a coarse flesh towel. In cerebral congestion the combined bath is sometimes used; here the patient is seated in a bath, the water being at a temperature of 110 deg., and a shower of cold water, either continuous or as a spray, is made to fall on his head.

The immersion of the entire body in a hot bath for a considerable time, say three to six hours, the water being maintained at an equable temperature, is useful in some cases of acute mania, and in ordinary insomnia; and the compulsory shower bath, given morning and evening, and lasting not longer than 25 to 30 seconds, is one of the most successful means of treating melancholia and some cases of acute mania when the physical health is good.

The application of alternate dry heat and cold has been found beneficial in some forms of spinal disease, and ingenious instruments have been constructed with the object of making the intervals between the application of the heat and cold as short as possible.

Finally, there are the innumerable numbers of medicated baths, in which the object is to induce the skin to absorb the various medicines held in solution; these kind of baths are very extensively used in hydropathic establishments, and prove of use in cases of gout and chronic rheumatic troubles.

* For much of the information contained in the sections "Electricity" and "Massage" I beg to express my indebtedness to Dr. Mills' "Treatment of Mental and Nervous Diseases." (English edition. Published by Baillière, Tindall, and Cox.)

### Mental Nursing.

NURSING EXAMINATION AT BERRY WOOD, NORTHAMPTON, COUNTY ASYLUM.

THE following have passed the examination at the end of the first year's training: Lucy Kealey, Annie Maidews, B. Jones, Rose Healey, T. Owen, S. Britten.

The following have passed the examination at the end of the second year's training: Henrietta Sensicle, Margaret Spencer, Minnie Bateman, Catherine Edwards, Elizabeth Rylett, Emma Hatton.

The following have passed the final examination at the end of the third year's training, and have received certificates of proficiency in mental nursing: Sarah Day (silver medallist), Charlotte Missenden, Fanny Iliffe, Clara Hatton, Emily Day, Fanny Daniel.

### District Nursing.

### V.—DISTRICT HYGIENE.

### Cleanliness, Food, Clothing.

With regard to the people themselves, district hygiene may be subdivided into cleanliness, food, and clothing. Cleanliness appears to be an acquired habit rather than an instinct in man, and many of the dirty ways so often encountered are not so much wilful delight in uncleanliness as want of training in better things.

### Daily Ablutions.

It is quite usual for the Saturday bath to be considered all the washing necessary for the family for a week, excepting the morning scrub of face, neck, and hands. Certain trades and occupations require greater cleanliness than this, it is true, but that fairly represents the ideas of the majority of the labouring classes on this matter. Public swimming baths are a boon to men and boys, also the public hot ones, but they are very little patronised by women.

Let the nurse be able to make a mother understand why daily washing of the whole body is so necessary to health, let her explain in as homely language as she will the effect of choked pores on lungs and kidneys, and so drive home the fact that the action of water on the skin is nearly as essential as water to drink. It is not only "respectable" to be clean, but necessary for present and future health. It is always one of the first difficulties encountered by all nurses in hospital or infirmary, this washing of dirty patients, and district nurses labour under greater disadvantages in every way.

### Difficulties Raised by Friends.

The friends are anxious, fearing the patient will be chilled or disturbed; the temperature of the room is often variable, and therefore chills may be given after the nurse's visit; the blankets, &c., are often wanting altogether, old coats and skirts taking their place; so obtaining personal cleanliness is a work of time and tact, especially if the patient is not ordered sponging by the doctor. A word of caution to district nurses is not to wash too much at first. It sounds most unorthodox, but it is better to cleanse by degrees, unless the medical man in charge of the case knows the method of the nurse's work. Most exaggerated statements are often made to the doctor by patients and friends unaccustomed to the process, and naturally he objects to undue exertion for his cases if he does not know how carefully it is done.

### Reforms Made Gradually.

The same applies to the cleaning of rooms; they must be done gradually, as confidence is gained in the nurse. The worst evils, such as soiled linen, coals, vegetables, &c., under the bed, can be removed at once, and all dirty utensils, pails, &c., cleansed and disinfected. But here again it is better to let the friends pack away their treasures themselves than for the nurse to attempt it. It worries the patient far more than the better ventilation improves his condition to have goods and chattels strewn about the room by a stranger, and put into places inconvenient for the family. There are many cases in which the nurse's help is gladly accepted, and it is always more satisfactory when she can put the room in order herself, but it is generally wiser for the friends to take their share; and nothing should be done without the consent of the principal member of the family, whether it be husband, wife, son, or daughter. There is always one ruling spirit, and any changes made without consultation invariably cause friction. Tact and observation are specially needed to avoid any collision and yet carry out the nurse's purpose.

### The Value of Water.

And, while enforcing cleanliness, it should not be forgotten that water is often a valuable commodity, both in town and country. When every drop has to be laboriously carried from the basement to the attics, often up five, six, or even seven flights of narrow, dark stairs, or brought from a distant pump or well in all weathers, the nurse will not lightly throw away half a basinful because she used too much lotion or put in too much cold water, as the case may be. This reason explains often what at first sight seems wilful neglect. Soap, also, costs money, and it would not be so frequently left wasting in the basin of water if the nurse realised that fact.

### Want of Thought.

These are some of the many details which only experience can teach. How few nurses understand at first that spilling water while emptying basins or filling kettles means that it will run between the badly-fitting boards of the floor, and stain the ceiling of the room below unless at once dried up, or that carelessly putting basins, medicine bottles, glasses, &c., which are wet underneath, on a polished table or chest of drawers, will leave permanent marks on what most likely is the best piece of furniture in the home. District nursing furnishes endless examples of the truth that

> Evil is wrought by want of thought
> More than by want of heart, and this is why it calls for the best workers, because so many interests are involved in nursing every case.

### Food.

Knowledge of physiology comes to the nurse's aid on this point, and enables her to speak from facts in answer to traditions. As a rule, the food of the poor is on mistaken lines from the beginning to the end.

From the day of its birth a baby is handicapped by traditional feeding. When all it needs is sleep it is made to swallow sugar and butter or gruel, and is fortunate if it escapes a dose of spirits when it evinces the discomfort caused by the unwholesome diet. If mothers would realise that Nature's way is best, and that their babies need no more than they can supply, there would be fewer cases of mammary abscess and convulsed infants.

### A National Question.

The question of feeding infants and young children properly is a national one, as it affects each generation. It is a herculean task, but each mother convinced of her mistakes, and willing to try the right way, forms a nucleus of further improvement. She must be made to realise that sopped bread and nursery biscuits are as useful food for an infant of two or three months as boiled sawdust, though excellent at six or seven months of age; that milk is essential up to twelve months old, with bread, broth, eggs, milk puddings, &c. It may be said poor people cannot afford such a diet, but except under special circumstances, when the family finances are at a low ebb, the ordinary labourer's wife could procure such food if she knew how and why to do so. In London eggs are a common article of food. Milk, and good milk, too, is cheap, and rice, sago, &c., can be bought for ½d. It is more trouble, doubtless, to make a special pudding for the baby than to let it share the family meal of potatos and greens fried in dripping, but it could be done. It is education that is needed.

### Infants' Feeding Bottles.

Absolute cleanliness of feeding bottles must be enforced by example and precept. Sickness and diarrhœa are often due to neglect in this detail. As the child grows older its diet is more varied, and so much can be done with rice, stewed bones, vegetables, &c., that the plea of cost is out of the question. But the mother so seldom goes beyond the frying-pan, and the extravagant chop and steak, except indeed on Sunday, when usually a joint of some sort is obtained—the poverty must be of the direst if the Sunday dinner is foregone.

(*To be continued.*)

### St. Michael's Home of Rest, Lyme Regis.

BY A TRAVELLER.

THE January number of THE HOSPITAL contained a short notice of the Lyme Regis Cottage Hospital, and it reminded me of another institution in that quaint town which is, in my humble opinion, of equal importance. This is the Home of Rest, founded especially for those who are "weary in well-doing" or rather from well-doing.

Lyme is a dear, sleepy little town, with inhabitants somewhat full of their own importance, yet too indolent to impress this upon visitors, unless greatly encouraged. Lyme seems to me the very ideal place in which to rest.

Energetic people can walk, play tennis, or ramble on the landslip, and scramble on the shore. But to the weary, to whom the chief pleasure of a holiday consists in absolute forgetfulness of the world and its worries, Lyme without these diversions, is a haven of rest.

Nothing to do, nothing needing to be done, leisure to sit, unmindful of time, on the walk which faces the sea, and the sea is strange in its contradictions, for it is restless and yet restful. From this warm nook, almost surrounded by cliffs, the tired worker can dreamily watch the life around, the bathers, the merry children, the glorious sunset, until nature asserts herself, and the pangs of hunger cause all footsteps to turn homeward.

St. Michael's Home of Rest for Gentlewomen has not such a beautiful outlook as the hospital, but it is not intended for the sick or for those confined to the house. Built in a narrow little street leading to the church, with its flat front and windows all in a row, it almost disappoints the newcomer until the door is opened. Then, in the front of us, rising in the centre of a square hall, is a beautiful old oak staircase with a balustrade on each side and broad shallow steps, the very things for weary feet. There is oak everywhere, but in some places it has suffered from the follies of fashion. When wall papers became popular splendid panelled walls were rapidly plastered and papered. Several rooms have been restored, but funds have not as yet permitted more.

The inmates—that sounds suggestive, but I can think of no other word—are absolutely free from any responsibility, the housekeeping, &c., being done for them, and, except for a few simple rules, they are able to come and go as they please. One of these rules, after dealing with punctuality at meals, &c., goes on, "and do all in her power to make the home happy." Those few words show the heartfelt wish of the founder, "To make people happy," is truly her motto, and to help those who are weary in body and soul seems her aim in life. The Home was started last year, and in its founder's eyes will prove its success when it is shown to be helpful as well as needful.

The sight of the Cottage Hospital made me think that as so many homes are provided for the sick, they are likewise needed for those who nurse, and I earnestly hope that some readers of THE HOSPITAL will obtain useful particulars by writing to the Founder, at St. Michael's Home. The terms are arranged to suit all purses.

### Nursing Classes at Bow.

IN connection with the Recreative Evening Home at Bow a nursing class has been instituted under the superintendence of Nurses Mumford and Waddell, who once a week teach the girls employed by Messrs. Bryant and May Bell's "practical nursing and ambulance." The members of the class have shown much interest in the instruction given, which is likely to be of great practical service to them.

### Sick Nurses in Italy.

BY A PATIENT.

THERE is not much to be said about native nurses in Italy, because, like the time-honoured "snakes in Ireland," there are none. The sick nurse, as such, is an unknown quantity, sufferers there being cared for by nuns, whose intentions are good, but whose knowledge is scanty.

After a time, from experience and experiments, they gather facts together for themselves, and of course from the beginning they know how to be strictly obedient to the doctor; a good starting point, but the doctor's instructions have to be very literal and minute, the comprehension of these good women not being of much account. As nuns they are not allowed to go about by themselves, and when one is required to nurse a sick person a messenger has to conduct the nun to and fro.

Being once taken ill in Genoa, I had to avail myself of the services of one of these good women as a night nurse. The "kitchen-maid," a somewhat brigand-like looking man, was sent for the Sister every evening; he, in company with about a dozen other menservants (well-conducted Italian women servants will not go out at night), had to stand outside the convent door until the hour struck and the nuns came out, whereupon each messenger pounced upon his particular nun and conducted her home. My nun was a thin, saintly-looking woman, while her conductor would have filled the *rôle* of a stage villian to perfection—they were an odd couple!

The Carmelites will supply one nun as nurse, but most of the other sisterhoods only send them out by twos.

As nurses they leave much to be desired. They have no idea of "tidying up," and they will use the same glass for medicine and lemonade, and will give the patient a pocket-handkerchief that has already done duty over a linseed poultice, with a splendid indifference to what appear to them the fads and fancies of the sufferer.

They are not particularly tender or sympathetic, for they have got so far in mortifying their own bodies that tenderness for the sufferer's is somewhat overlooked; but they are patient, carry out instructions, and never drink. They are unrelenting talkers, miracles and the saints being their chief subjects; it is the merest detail that an ordinary English traveller only understands about a tenth part of what they say. They never take off their hoods and veils, so that the patient has none of the cheery freshness of the English nurse's pretty white cap, these cowled figures being awe-inspiring rather than cheering.

Their voluminous flannel habits are fine lurking-places for germs; they never wear aprons, though they tuck their sleeves into smaller linen ones, with a band at the wrist.

The medicine bottles are not neatly labelled with a gum ticket, as in England, but have a long, comet-like piece of paper fastened to their necks; these papers frequently come off, and as often as not they serve to light a candle with (which is one point in their favour), but, in spite of their disappearance, the patient seems generally to get the right dose. It is wonderful that accidents so rarely happen, for much seems to be left to chance, and to those usually fatal things, "good intentions."

### Sunderland Infirmary.

PRESENTATION OF CERTIFICATES.

THE nursing at Sunderland Infirmary has made steady progress during the last four years, and on January 23rd certificates were presented to the following probationers on the completion of their three years' training: Emily T. Bone, Adeline Fyfe, Eleanor Smith, Dora K. Mohun, Flora Thorpe, and Henrietta Frater. Nurse Smith received a special certificeate and a prize of books for doing best in the examination and in ward duties. A special prize of a gold medal, offered by Dr. Squance for the highest number of marks in the examination on hygiene, was obtained by Mary McDonald.

### Our Examination Questions.

A number of replies to our question respecting the nursing of diphtheria have reached us. Many give excellent instructions as to the precautions needful, the nourishment of the patient, the proper temperature of the room, &c. Whilst some papers offer very good directions as to nursing details, others occasionally fall into the error of suggesting medical treatment. However, this fault is happily far less common amongst our competitors than it was even one year ago. Our readers seem to understand that our examination questions deal entirely with the nursing of patients, and are meant to be helpful to all who are engaged in caring for the sick. Each paper mentions the need for isolation of a person suffering from diphtheria, but we do not quite understand why nearly all should speak as if a particularly large room were an absolute necessity. If a nurse finds her patient already lying ill in bed in an apartment of moderate dimensions, she will naturally put and keep the chamber in perfect nursing order. Unless the doctor wishes a change made no nurse would dream of removing the invalid out of the already-infected room, therefore it is obviously absurd to describe an ideal sick room, and to speak of putting the sick person to bed in it as a regular preliminary to nursing him. When such a serious disease as diphtheria has secured a victim, the latter seldom waits for the nurse's advent to decide where he will go to bed. Opinions as to the amount and kind of nourishment required seem to vary enormous one writer deciding that food should be given every two, and another every four hours. We trust that the latter does not carry her theory into practice. One person speaks of exclusively dieting with milk and eggs; another omits the all-important question of nourishment entirely !

We are particularly pleased to see how well disinfection is described by many corresopndents, and we congratulate them on the thoroughness they advocate. In The Hospital of January 27th there is an excellent article on the "Pathology and Treatment of Diphtheria," which we commend to the careful consideration of our readers.

Examination Question.

Describe the nursing of a case of diphtheria, and the special precautions necessary.

Prize Answer.

In nursing a case of diphtheria, the patient and nurses should be isolated, and all vessels, *i.e.*, feeding cup, spoon, plate, &c., be kept exclusively for the patient's use. Old linen should be used instead of handkerchiefs, and this should be burned immediately. All the soiled linen should be placed in carbolic lotion, and directions given to the laundress to have it washed separately.

The patient should be kept warm in bed, and a light nourishing diet given frequently; the temperature taken every four hours as well as the pulse and respiration. The nurse should watch for laboured or noisy breathing, and report at once to the doctor, whose orders for treatment, whether local or otherwise, should be strictly obeyed.

The patient may become suddenly worse owing to the spread of the diphtheretic membrane into the larynx, and it may be considered necessary to perform tracheotomy, in which case, after the operation, care will be needed to keep the tube in position, and to keep the inner tube clear. It may be necessary to take out and clean the inner tube every half hour, but the frequency will, in a great measure, depend on the amount of expectoration. In any case it ought to be cleaned not less often than every two hours for the first twenty-four hours. A strong soda solution will be found a very efficacious agent for freeing the mucus and shreds of membrane from the tube, and if the clean tube is then dipped into a little glycerine it will slip readily into the outer tube.

The necessity for operation has shown the patient's condition to be a most critical one, and therefore the utmost care and watchfulness is required from the nurses. Bronchitis is a troublesome complication, so that it is necessary to keep the atmosphere warm and of an even temperature.

A steam-kettle will most probably be ordered, but the doctor will give full directions as to his mode of treatment.

Later on signs of paralysis must be watched for, the return of food, especially fluids through the nose, being one of the first symptoms. The patient must be kept very quiet, as the heart is in a very weak state, and very slight exertion may produce faintness and sudden death.

After the doctor pronounces the patient free from infection the room and all its contents must be fumigated, the walls repapered, the ceiling whitewashed, mattresses, pillows, blankets, carpet, and all woollen materials sent away to be stoved.

E. A. Anderson.

Examination Question for February.

How would you give "first aid" to a patient who had sustained a compound fracture, and in such case, what would you get ready for the surgeon in a private house or a cottage?

Answers must be sent in by February 17th, written only on one side of the paper, accompanied by the writer's full name and address, and directed "Nursing," care of Editor of The Hospital.

### The Nursing of Ovariotomy Patients.

By J. Lawson Dick, M.B., C.M.Edin., Resident Surgical Officer, Clinical Hospital, Manchester, late Junior Assistant to Professor of Midwifery, Edinburgh.

### I.—THE OPERATION.

From the day when even the boldest surgeons deemed ovariotomy an operation unworthy a place in surgery, to the day when Sir Spencer Wells writes, "We may now confidently calculate upon an average mortality of not more than three or four per cent.," is a stride of which surgeons may well be proud. Doubtless, the greatest factor in producing such a change was the introduction of surgical cleanliness after the teaching of Sir Joseph Lister and his school. But this is, after all, only one factor. The minute attention to detail in the preliminary treatment, actual performance of operation, and after-treatment must ever hold an important place in making ovariotomy a safe and justifiable operation. The preliminaries and the actual performance of the operation are, as a rule, carried out under the immediate directions of the surgeon, but not infrequently the nurse in charge of the case has the after-treatment virtually in her own hands. A short summary then of the more important points in the treatment after ovariotomy may prove useful in what must always be a trying and anxious time to the special nurse.

The After-Treatment.

The operation having been performed, and the binder fastened, the patient should be carried back to bed without delay. If a sufficient number of persons are present, this is best done by lifting her on the sheet on which the operation has been performed. If only two or three are present, one may take the shoulder and head, whilst another places her hand under the pelvis and lower limbs, and so the body is raised. Before the patient recovers from the chloroform, the sheet on which she was carried back to bed must be removed, and the night-gown, which was tucked up round the chest before operation to prevent its being soiled, must be pulled down. She must be covered with warm blankets, and have two or three hot bottles at the side and one at the feet. When the patient regains consciousness, there is sometimes great restlessness, and it is necessary to see that at no part do the hot bottles come in contact with the skin during the constant movements, otherwise a scald might result. After the patient has been carefully arranged in bed the temperature should be taken. As a rule it will be subnormal, and indeed, for half an hour or so, the patient will look rather collapsed. This fact calls for careful attention, in order that stimulant, either by enema or hypodermically, may be given if required. The pulse is an excellent guide. Mere rapidity does not mean much, but irregularity either in the length of the intervals between the beats, or in the force of the beats themselves, should always be a cause of anxiety. If there is chloroform sickness, care should be taken that the patient does not strain unnecessarily. During the effort of vomiting the nurse should place her hand gently over the abdomen to lessen the strain on the wound. She should also watch that with the movements of the patient the dressing does not slip up, and expose the lower part of the wound, this being a fertile cause of stitch abcesses. It can be avoided by having a large square piece of gauze placed over the boracic lint or other dressing next the wound, and sealing the edges of this to the skin with collodion.

### The Presence of Pain.

For the first twenty-four hours a good deal of pain is experienced, and this raises the vexed question as to whether or no morphia should be given. As a rule it is best to avoid its use entirely. Apart from the fact that morphia conceals the true condition of the patient, it is most essential in all laparotomy cases that the excretory organs should be in good working order. Now, morphia tends not only to retard the urinary secretion, but also to paralyse the bowels, a condition which greatly favours the occurrence of tympanitic distension of the abdomen on the second or third day, and the continued non-passage of flatus by the rectum. Moreover, the pain after ovariotomy, though severe at first, tends to disappear, and the patient can be comforted with the assurance that at the end of twenty-four or thirty-six hours it will probably be quite gone. While this is the rule, however, exceptions will now and then arise, when, on account of the restlessness and actual suffering of the patient, it will be well to give a quarter-grain of morphia hypodermically. Not infrequently trouble is experienced in getting the patient to pass water. Neither before nor after operation should a catheter be used unless unavoidable. If on the evening of the day of operation the patient has not passed water, it will be well, after she has had her hands and face sponged, to suggest to her that she should do so. If unable to do so, the suggestion may be repeated during the night. The application of hot fomentations to the pudenda is often useful. In any case, the actual use of the catheter may be postponed till morning. If the catheter must be used, let it be a No. 8, taken out of weak carbolic lotion and dipped in carbolic oil. Cleanse the vulva, and pass the catheter, trusting rather to sight than to the sense of touch in doing so. In the great majority of cases, however, the catheter can be perfectly well done without. The surgeon on his next visit, after looking at the temperature, will ask two questions as a rule—firstly, if the patient has passed water, and, secondly, if flatus has been passed by the rectum. The free passage of flatus by the rectum is a most satisfactory symptom, and is one which the nurse should always be on the alert for. Supposing no flatus has been passed by, say, the middle of the second day, it will be well to pass the rectal tube, which often favours its descent.

### The Temperature.

The temperature should be taken every two hours for the first week. As already stated, immediately after operation the temperature is not infrequently subnormal. In the course of three or four hours, however, reaction sets in, and the temperature rises during the course of the afternoon and evening to 100 deg. Fahrenheit or 101 deg. Fahrenheit, or even more. The continuance of subnormal temperature or a sudden fall, especially with symptoms of collapse, is always a sign of danger.

*(To be continued.)*

### Nursing in the United States.

### A NEW WORK OF TRAINING "ATTENDANTS."

Any new movement that is successful is quite likely to be followed by the query, "Why was this never done before?" Whatever opens another branch of wage-earning has a social and economic value which helps to solve the difficult problem of the unemployed. When the new departure involves training it is doubly valuable, for unskilled labour lies at the root of industrial life sapping its strength.

The proper care of the sick has gradually evolved the splendid enginry of the modern hospital, and its necessary accompaniment of a training school for nurses. But every physician has felt a gap in trying to establish suitable attendance for that class of patients who do not need the services of a trained nurse, and yet require more care than can well be bestowed by members of the household. The aged, the chronic invalid, the convalescent, the crippled, the young children—who shall care for them in busy families? Trained nurses are expensive; besides, these cases do not require professional skill, but they do need constant care and some one at hand to do the thousand little offices which mitigate the weariness of invalidism.

Another want has been experienced in the sick-room. A nurse by day and another by night, besides a physician, involves an expense too great for many a household, where, nevertheless, they are greatly needed. The sick one's relatives and friends are not always available to relieve the nurse when she goes for a breath of fresh air or takes the necessary hours of sleep; besides, they cannot always be trusted, and their inexperience may do positive harm to the patient. To fill these gaps a wise step has been taken by the Massachusetts Emergency and Hygiene Association, which has its headquarters in Boston, but branches in other cities and large towns. This new venture is known as training classes of women as "attendants."

The idea originated in Brooklyn, New York, where the work was first carried on, but in November, 1892, it was reduced to a practical permanent basis in Boston. The above association secured the services of a trained nurse (Miss D. H. Kinney) as instructor, for she must know a great deal more of a subject than ever she demands of her pupils. She is a graduate of the Training School for Nurses at the Massachussetts General Hospital, Boston, and has carefully worked out a schedule of instruction, adding to great clearness in teaching, much enthusiasm, and good common sense. The classes number from fifteen to twenty pupils.

The course includes thirty lessons on specific duties in the sick-room, ventilation, taking temperature and pulse, preparation of poultices, giving of baths, serving of food, and the many small devices for lessening the discomforts of convalescence. Some knowledge of physiology is a necessary basis, and, as far as it goes, the instruction is intended to be thorough. Pupils take notes at each lesson, and are frequently subject to a "quiz" to freshen the memory. At the end of the course the class is examined by a physician of standing, either orally or by writing, as he prefers. To those who pass satisfactory examinations, certificates of their proficiency are given, and by paying a fee of $2 a graduate may be registered at the Bureau of Nurses. They pledge themselves to ask only $7 a-week during the first year.

Women entering these classes must be over twenty years of age, intelligent enough to take notes and write out an examination, and must show some fitness for the work. Many applicants are rejected as unsuitable at the outset, and others, who betray inability to grasp the ideas as the lessons progress, are quietly advised to withdraw. The tuition fee is $6. Some classes are held in the evening to allow wage-earners to take the course without relinquishing their daily work. It is evident that this work of "attendants"

demands less intelligence than is required in a training school for nurses, and is available for many who could never become trained nurses. These "attendants" are not nurses, and must not be confounded in the popular mind with graduates of reputable training schools who have given two years of study and rigorous preparation. Confusing these two lines of work, which are quite distinct, each admirable in its scope, would tend to lower the standard of a nurse's salary, and cheapen a profession which it has taken two decades to raise to its present dignity.

Many physicians are warm advocates of these "attendants," while a few are following the new step with doubts as to its permanent value. The fact that of the fifty attendants now on the list at the Boston Registry of Nurses, only five are without places, indicates a demand for just this kind of helpful service.

During the winter of 1892-93 Mrs. Kinney had in Boston three daily classes, two evening classes, one class in the domestic science department of the Young Women's Christian Association, and one class of ladies for home helpfulness. She also conducted a successful class in Worcester, Mass., and in the New York cities of Syracuse and Auburn. Thus the idea is gradually spreading through the country, providing limited service in the sick room at moderate rates, and giving another avocation to wage-earning women.

### Women's Work.

### A VOLUNTEER MEDICAL STAFF CORPS FOR WOMEN.

By Ethel Stokes.

Before giving an account of the proposed Women's Medical Staff Corps, it will perhaps be best to enumerate briefly the general ideas which have led to the formation of the scheme.

It was at first proposed to form a Volunteer Rifle Corps for Women, in the hope that it would prove both a school, where women might learn to fulfil the more active duties and responsibilities of citizenship, and, at the same time, a field for the useful exercise of such civic capacity as they already possess—a capacity at present almost entirely disregarded by the community. As the scheme grew, however, we saw that women could render the State a far greater and less disputable service, if they could form an efficient body of doctors and nurses, under military discipline, capable of acting in conjunction with the Army Medical Staff and Corps, and the Volunteer Medical Staff Corps, whenever those bodies were called into action. The alteration in plan seemed equally justified from the standpoint of women themselves; for it is a department of the public service for which they are pre-eminently fitted by nature and by previous training; while, as an organised body, it would furnish the same, if not greater, advantages of discipline and public axperience.

There is ample room for another medical corps, and we believe that women's assistance will be welcomed both by the authorities and by the Men's Volunteer Corps; for the Army Medical Corps is by no means sufficient to supply the medical aid required in case of war, a fact which is practically acknowledged by the Department in the placing of the V.M.S.C. in the 1st Army Corps, with headquarters at Netley, on the outbreak of war. The Volunteer Corps numbers less than 2,000 men, and if this number is compared with the strength of our regular and volunteer forces, it will be seen to be very inadequate in time of war.

Our reasons for believing it better to establish a corps under military discipline rather than to form a volunteer band of auxiliary army nurses are: First, that the corps, if formed, will be able to take care of itself in war, in exactly the same way as any other body engaged in the service, being prepared to march, encamp, and perform all duties incident to a campaign, whilst a body of hospital nurses would always have to be provided for by others, or would fare ill. Second, if the corps fulfils the conditions as to numbers and efficiency which the War Office may appoint, and earns the Government grant, they will, in return, be able to guarantee to Government that a certain percentage of them shall be ready for service on the outbreak of war, whereas those responsible cannot estimate what amount of aid they may on any occasion receive from the Red Cross Sisters and other heroic volunteer nurses, who, besides, have not the advantage of military discipline. Third, from the point of view of women themselves, whose interests gave the first impulse to the idea, a corps under military discipline, and drilled as is required by the regulations, would provide them with a training which it is impossible for them to get elsewhere, and which would, we believe, prove of the greatest service.

Those who are interested in the scheme are thus endeavouring to establish a women's medical corps on the following lines, which can only be sketched in broadly, for in a new undertaking like the present it is difficult to settle details without some experience in practice. The discipline and first training of the corps must necessarily be that laid down in the "Manual for the Medical Staff Corps," published by the War Office; and those who join without any previous knowledge of nursing, &c., will be instructed and examined as it requires. The training given will thus not be exactly that of a hospital nurse; it will, of necessity, embrace some points which she does not require, such as a certain amount of company drill, and a modified course of musketry instruction; there will be tactical practice also in hospital encampments, &c.; and in some other departments the training will not be so detailed as a nurse's, nor so much concentrated within a certain period. This latter must not be supposed a loop-hole by which unthoroughness and superficiality may escape and pass muster; but, from the nature of the case, we cannot hope that all our volunteers, most of whom will probably be engaged during the day, will be able or willing to give up three years entirely to hospital work. The training must be carried on in exactly the same way that is adopted by the V.M.S.C., which has produced a most efficient body of men.

We hope that the scheme will interest many of the noble-minded women who engage in nursing as a professsion. Some, perhaps, may be able to join us, and add their knowledge and experience to our as yet untutored zeal. We have reason to hope that if we could provide forty or fifty nurses, trained in the discipline above described, we should always be able to procure work for them, in places where nurses, under military discipline, would be far more useful than the ordinary hospital nurse. We should thus be providing employment for more nurses, and they, in their turn, would, from the permanence of their work, add greatly to the strength and continuity of the corps.

In the course of a few weeks a meeting is to be held, of those willing to promote the scheme, for the discussion of the best way of commencing work; after which we hope the Volunteer Medical Staff Corps for Women will be raised to the realm of accomplished fact.

### Where to Go.

Chelsea Town Hall.—At a quarter-past eight on February 6th, and Tuesday evenings following, Mr. Churton Collins will lecture on the "Poetry of Robert Browning."

Clapham Maternity Hospital, 41 and 43, Jeffreys Road, Clapham.—Annual meeting at half-past three on Wednesday, February 7th.

Trained Nurses' Club, 12, Buckingham Street, Strand.—A course of six "Nursing Talks" will be given at this club on Tuesdays, at three o'clock, commencing on February 13th. Fee for the course, seven shillings.

☞ For Modern Teachers, Book Reviews, Everybody's Opinion, and Reading to the Sick, see page clxxix. et seq.

### Modern Teachers.

### MRS. SARAH GRAND.

Mrs. Sarah Grand is the "boom" of the hour. There have been a good many of these "booms," but few of them maintain their popularity beyond a year. Everybody has read the "Heavenly Twins," so we won't discuss that alone, but consider Mrs. Grand's doctrines as gathered from various of her works. We say doctrines advisedly, because Mrs. Grand considers herself a teacher, who selects the form of her expression according to her audience. For "earnest women" it is best to write essays, as that on "Morals and Appearance" in the "Humanitarian"; to weaker vessels you preach by means of the "novel with a purpose," or, in Mrs. Grand's case, with several purposes, for the "Heavenly Twins" has purposes enough to overload at least five novels. Mrs. Grand says, "It is the way in which the argument is presented that rouses us." "When you deal with the multitude you must think of it as an emotional rather than an intelligent body, and however capable you may be of appealing to its head, you need have small hope of success, unless you can also touch its heart." And what is it after all that Mrs. Grand is so anxious to teach, and to cause all women to teach? Firstly, she seems to desire that all women should "hold opinions," even "though they never express their opinions further than by letting them be known," for "everyone is not eloquent." In spite of this concession to their non-eloquence, how are they to "let their opinions be known" without expressing them? We are told that "the woman who spends hours considering the points of a costume in which she shall be coquettishly alluring to men in a ball-room is an animal lowering herself by the endeavour; but the woman actuated by noble purpose, who selects a costume which shall help her to please by her appearance those whom she hopes to convince by her arguments, is worthy of admiration." Mrs. Grand wishes women to missionise without ceasing, and for that purpose to cultivate every possible charm of face, dress, and manner so as to lead the enemy, man, into captivity and the forced adoption of "opinions." "If you wish to convince, remember that your argument is a pill which must in any case be bitter to your opponent, and when you offer it to him (Mrs. Grand's 'opponent' always 'him') silver it with a smile," and in this the sex must persevere, not "by forcing their opinions with brutal insistance" (for "persistance, not insistance, wins the day"), remembering that "women are too prone to strife," but feeling encouraged by the knowledge that "women are richly endowed with the irresistible magnetic force, which can be increased by cultivation, and by the help of which there is nothing we could not compass if we chose." With much of this we can find no fault. It is for the weal of the majority that women should be gentle, prettily dressed, and nicely mannered, and it is equally certain that those who hold opinions are most likely to be listened to respectfully if they fulfil these conditions. But what opinions does Mrs. Grand hold, and are they the sort of opinions that we want our wives and daughters to lose no opportunity of bringing to the notice of the "opponent"? This word opponent as applied to men seems to sum up most of Mrs. Grand's philosophy. Women are to be ever on their guard. Men, or rather husbands, are not co-workers for good, but insidious enemies, if not brutal conquerors, according to her. We do not think this arrogant stone flinging wholesome for women or girls, and so far as the "Heavenly Twins" are concerned, we think it a most unsuitable book for girls of limited experience. It is enough to breed dread and suspicion of they scarce know what. Those whose lives are passed perforce among vicious people, or whose work necessitates contact with such, are unable to escape from knowledge of the disgusting consequences of vicious lives, and do not require Mrs. Grand's teaching. For those sheltered from such knowledge what good can come of filling them with disgust and suspicion of every man, however virtuous he may outwardly appear. Such a novel as this, with its deceptive title, passes into many an unsuspecting household and its horrors are unfolded to the innocent girl, who after such a book must cease to be innocent for ever.

Mrs. Grand, next to man, seems to abhor "the early Christians," to whom she attributes all manner of narrow-minded bigotry, and about whose history she seems but imperfectly informed. We always believed that they revolted against the tyranical dogmatism of the Jews, who subsequently persuaded their conquerors, the Romans, to assist in their persecution. Mrs. Grand has it that it was in opposition to "pagans" of Greece that the first teachings of the Christian* religion were formulated. "They perceived that the pagans possessed in the refinements of manner, and distinction of appearance they cultivated, an argument in favour of their system which there would be no resisting at close quarters if it could not be discredited, the Church, therefore, taught that such graces were snares of the devil. "Cleanliness was a virtue among the pagans; the Christians condemned cleanliness of person, made dirtiness a virtue, and taught that the longer you went without washing the fitter you were for the Kingdom of Heaven." We think the early Christians' "system" had a stronger foundation than Mrs. Grand suspects, or it would not have survived the "pagans" for nearly 1,900 years. Such diatribes are so obviously untrue that we only cite one as a specimen of Mrs. Grand's prejudice and inaccuracy.

"The Sere and Yellow Leaf," in the *Pall Mall Magazine*, is a dull caricature of the "Society mamma." She must be the Society mamma of some wealthy provincial town, and certainly not one of the "classes," whose mammas model themselves very strictly on Sandringham lines, and as closely as possible imitate H.R.H. and the ever-adored Princess of Wales in the careful manner they "raise" their daughters. We have thought it right to protest against Mrs. Grand's crusade, for just now no magazine editor feels up to date if he has not some of her wares to advertise. She made people "sit up" over her "Heavenly Twins," and they will go on buying her in the hope of "sitting up" again. And if she oblige them by continuing as she has begun, they will peruse many an unpleasant story as of Zola and George Moore, plus a "purpose."

* Essay on "Morals and Appearance."

### Midwifery Training at the Bristol Royal Infirmary.

At the end of 1892 it was considered advisable to make a new departure at this hospital by utilizing the Eastern Maternity Charity for training those of the staff who wished to fit themselves for monthly nursing. The experiment has proved an unqualified success, the course of training being popular with the nurses themselves, whilst their attendance proves a great boon to the patients. Nurses undergoing the midwifery course attended 290 poor women in their own homes. Puerperal complications have become reduced to a minimum, and convalescence has been more rapid since the employment of the hospital nurses. They each attend on an average from 50 to 70 cases, and at the end of the course they present themselves for examination by the Board of the Obstetrical Society of London. At the recent examination all the candidates passed with credit. The course of training at the Bristol Royal Infirmary may therefore be considered a most complete one.

### Appointments.

Worcester General Infirmary.—Miss Mary Herbert has been appointed Matron of this infirmary. She was trained at St. Thomas's Hospital, and was afterwards Sister of Elizabeth Ward, night superintendent, and assistant matron, and we wish her every success in her new work.

### The Book World for Women and Nurses.

[We invite Correspondence, Criticism, Enquiries, and Notes on Books likely to interest Women and Nurses. Address, Editor, The Hospital (Nurses' Book World), 428, Strand, W.C.]

Hints on Home Nursing. By Dr. R. T. Halliday (Published by Hay, Nisbet, and Co., London and Glasgow. Price 1s.)

We are decidedly at a loss to know why this book was ever written. In the preface the author says it is to provide a text-book for the St. Andrew's Ambulance Association, and hopes it "may prove of value for guidance and reference, not only to those attending the classes of the above association, but to all who may have at any time the care of the sick. Even for the professional nurse there is much to mark and inwardly digest." We fear we must entirely disagree with this very sanguine hope. Those who "have the care of the sick" without training, and who depend on this manual, will find they have much superfluous matter to wade through, whilst for the professional nurse there do not appear any hints that will be useful to her, and where the teaching differs from that given in a first-rate nurse-training school we advise the nurse to adhere to the latter. Amongst the superfluous matter referred to we would include the verses of poetry at the beginning of each chapter, besides other verses and maxims and long quotations from the *Times*, *Lancet*, *Chemist and Druggist*, and from various writers. The author, not satisfied with his own definitions, adds long quotations, as, for example, in the case of "Disinfection."

The author very rightly insists on the necessity for keeping the patient quiet and not annoying him with irritating noises, and gives minute instructions about putting coal on the fire and spreading sand on the hearth; also no clock is to be allowed in the room lest the ticking should irritate. After all this it is a surprise to the reader to find that a chatelaine, consisting of jangling chains, with scissors, forceps, &c., attached, is recommended to the attendant, "as is done by nurses in hospitals"; but surely the author knows that these objectionable chatelaines are discarded by true nurses.

We think the author makes a mistake in recommending so many appliances, so that the pages of this manual resemble an "illustrated catalogue," and in the case of the "literary machine," we are further told "they have proved serviceable to all classes, including Royalty. Our Queen conveyed such a machine to Germany for the use of the Emperor Frederick in his last illness, and the accompanying illustrations show them in use, in the one case of the late Rev. Charles H. Spurgeon and in the other of the Queen of Italy."

Surely after telling us to keep the patient's room at a temperature of 60 deg. to 65 deg. F., the author can hardly believe that ice placed in a pitcher covered with paper "may stand in the room overnight without much appreciable melting"? It is amusing to find, under the head of "Infection and Disinfection," that in advocating the hanging of a sheet soaked in disinfectant solution, *e.g.*, sanitas, carbolic, or chloride of lime, in the case of the latter it is recommended to use an "old sheet." Now as an "old" sheet has almost invariably holes, more or less numerous, and as the object of the sheet is, says the author, "to prevent germs finding egress from the sick room without encountering the action of the disinfectant," it will at once be apparent to the least experienced person what a doubtful protection such a sheet will be! How can we agree with the author that "vaporisers" of carbolic, creosene, &c., "keep the air of the room pure," for if enough disinfectant were used to effect this, the life of the patient would surely be endangered. Plenty of fresh air from outside is the best purifier, and a far less costly one too. We are surprised to find that Condy's fluid is recommended amongst the substances with which a patient's skin is to be "occasionally rubbed in scarlatina or measles," and hope the unwary attendant will first inquire whether the patient would like to be transformed into a gipsy or a "darkey." Excepting this precaution there are no directions as to the proper disinfection of the patient before he can be allowed to mix with other people with immunity, which is certainly as important as the disinfection of the room. The printing and binding of "Hints on Home Nursing" do not strike us as more commendable than the matter contained in this very cheap volume.

The Nurse's Dictionary, compiled by Honnor Morten. (Scientific Press, 428, Strand. Price 2s.)

A second edition of this handy little volume, the "Nurse's Companion," as it might be fittingly entitled, has recently been issued by the Scientific Press, and will doubtless command an extensive sale. It has been corrected and also enlarged, the compiler tells us in a concise little preface. Certainly it contains a wonderful amount of information.

---

### REVIEWS OF THE MONTH.

The Humanitarian for January contained an interesting article, embodying the views of Sir James Crichton Browne on a social question. The changed and changing character, in many respects, of the struggle for existence comes prominently before all thoughtful minds in these days; no less a struggle, nay, keener, year by year, but rather now a matter not so much of the survival of the fittest as "the multiplica- of the unfit." And this because "in social progress the struggle for existence becomes, in certain directions, a surrender, not of the feeblest, but of the strongest and the best. A recognition of the obligations which man owes to his fellow-men, and the promptings of 'love's divine self-abnegation,' impose restraints on some of the competitors, who, instead of forcing their way to the front, as they are well able to do, stand aside and allow themselves to be beaten by those less fitted to survive." This is demonstrated by the fact that while medical science in its latest developments has enormously diminished the general death-rate, it has increased amongst men above the age of 35 and women over 45, the result, this, of the survival of the unfit, and the consequently increased strain on the energies of the stronger minority.

Arguing from analogies in the natural world, increased co-operation would seem to carry with it a solution, in some measure, of the problem, and State Socialism aims at making the struggle for subsistence less and less the concern of the individual and more and more that of the State, thus removing some of the burden from the human unit. But the conditions under which such reform of the social state is to be obtained, according to some of the schemes put forward, would be dear at any price; the extermination of family life in its best and purest sense, is threatened, when children will grow up to feel that from the cradle to the grave they are indebted for all their needs to the State rather than to their parent's love and care, when the duties of parents will become non-existent, and parental responsibilities will come to be gladly shifted on to the State. Such is one side of the picture. But Sir James Crichton Browne gives us another. In his opinion, with which we are entirely at one, such a view leaves out of calculation the highest side of human nature, and but reflects the persistent gloom, the prevailing disease of the present day, which is surely but a temporary phase. We cannot believe that we are to become mere mechanical items in a State autocracy. With an increased reverence for the State, family ties should strengthen and grow in the best sense. "Evolution," says Sir James, "is progressive," and a dead level of uniformity cannot then be the ultimate condition of humanity. Let us rather believe with Sir James that "man, to a large extent, controls his own destiny, and may, if he will, rise out of the prevailing pessimism and climb to heights of sentiency not yet attained."

### Everybody's Opinion.

[Correspondence on all subjects is invited, but we cannot in any way be responsible for the opinions expressed by our correspondents. No communications can be entertained if the name and address of the correspondent is not given, or unless one side of the paper only be written on.]

### MIDWIVES' REGISTRATION.

DR. HUGH WOODS, Master of Midwifery, writes: Dr. Alderson approves of some kind of registration of midwives; but apparently not of the only registration which has ever been or will ever be proposed. A "midwife" is in no sense a nurse. She has a house of her own, and lives there, going out to visit patients, just as a doctor does. She has no legal status more than a bone-setter. She is simply a woman who, not being qualified to practise as a doctor, avails herself of the liberty accorded to all quacks, and assumes the rôle of a female obstetric physician. Midwives are, as a class, with a few exceptions, greatly inferior in every respect to "monthly nurses." Now as to legislation. If midwives are registered by Act of Parliament, they must and they will have a position as regards obstetrics similar to that which dentists have as regards dentistry. That is to say, they will be obstetric specialists while doctors are merely obstetricians, out of respect to existing privileges. Now, sir, at this stage of progress in the obstetric art, to relegate it into the hands of those who, in the middle ages, made parturition horrible, can hardly be condemned too strongly. There are far more than enough medical men to do all the obstetric work in the country, and it is only by mean parsimony that this is prevented. Doubtless a Midwives' Registration Bill would justify the State in providing inadequate attendance, as is now done, for parturient paupers; but enlightened papers like THE HOSPITAL have always maintained that the very poorest ought to be provided with medical attendance in their sickness as good as that accorded to the rich. Dr. Alderson's Registration Bill is to place midwives "under the control and supervision of medical men." How? But, sir, the serious matter is this: Every registered midwife will be in a position to act as obstetric assistant to a doctor without subjecting the latter to a charge of "covering" unqualified practice. This is the object of registration of midwives. It is intended to supply wholesale to the working and middle classes the gratuitous services of "registered midwives," officered by West-end consultants. In this way the consultants hope to oust general practitioners from obstetric practice, as they have done by means of hospitals (mis-named charities), from medical and surgical practice. But the general practitioners are on the alert, and the moment midwives are registered every sixpenny dispensary in London will be provided with a female obstetric assistant. The "unqualified assistant" will have obtained a legal status so far only, however, as regards the female sex. Dr. Alderson knows that the association of which he is president is pledged to resist all registration of midwives to the uttermost unanimously, himself excepted. Dr. Alderson talks of "the qualified and competent midwife." As well talk of the qualified and competent bone-setter. Why not register the latter after six months' training in bone-setting, keeping him under the "control and supervision of the medical profession?" This attempt to degrade the practice of obstetrics is a disgrace to our profession. But I speak that which I know when I say that it is in the ranks of the medical profession that unqualified quacks of all descriptions find their main, though often secret, support. Midwives, like other unqualified practitioners, are yearly causing more deaths than many of our dreaded diseases; and if they are registered they will have a legal right to do it. Space forbids my showing that registration alone is a failure, that nearly half the dentists now in practice are unregistered, and an enormous proportion of the doctors. Registration of midwives would do incalculable harm. It can do no good.

### For Reading to the Sick.

**Motto.**

I myself had been happy, if I had been unfortunate in time.

**Verses.**

Pain's furnace—heat within me quivers,
God's breath upon the fire doth blow,
And all my heart in anguish shivers,
And trembles at the fiery glow;
And yet I whisper, "As God will!"
And in His hottest fire hold still.

He comes, and lays my heart, all heated,
On the bare anvil, minded so
Into His own fair shape to beat it,
With his great hammer, blow on blow,
And yet I whisper, "As God will!"
And at His heaviest blows hold still.

He takes my softened heart, and beats it;
The sparks fly off at every blow;
He turns it o'er and o'er, and beats it;
And lets it cool, and makes it glow:
And yet I whisper, "As God will,"
And in His mighty hand hold still.

Why should I murmur? for the sorrow
Thus only longer lived would be;
It's end may come, and will to-morrow,
When God has done His work in me.
So I say trusting, "As God will!"
And trusting to the end, hold still.

He kindles for my profit purely,
Affliction's glowing, fiery brand:
And all His heaviest blows are surely
Inflicted by a master hand:
So I say, praying, "As God will!"
And hope in Him, and suffer still.

*The Changed Cross, &c.*

**Reading.**

A while ago I passed
Where every step seemed thornier and harder than the last;
Where bitterest disappointment and inly aching sorrow
Carved day by day a weary cross, renewed with every morrow—
The heaviest end of that strange cross I knew was laid on Thee;
So I could still press on, secure of Thy deep sympathy.

—*F. R. Havergal.*

By His fire God can kindle the smallest lamps to His glory, making them like the golden candlesticks that burn before His throne.—*Jeremy Taylor.*

There should be no greater comfort to Christian persons than to be made like unto Christ by suffering patiently adversities, troubles, and sicknesses, for He Himself went not up to joy, but first He suffered pain; He entered not into His glory before He was crucified.—*Book of Common Prayer.*

Who doubteth but God can bring us to Heaven through adversity and suffering? We see many rivers, but we know not their first spring and original fountain; yet they have a beginning. When ye are come to the other side of the water, and have set down your foot on the shore of glorious eternity, and look back again to the waters and to your wearisome journey and shall see, in that clear glass of endless glory, nearer to the bottom of God's wisdom, ye shall then be forced to say, "If God had done otherwise with me than He hath done, I had never come to the enjoying of this crown of glory." It is your part now to believe, suffer, and hope and wait on; for I protest, in the presence of that all discerning Eye, who knoweth what I write and what I think, that I would not want the sweet experience of the consolation of God for the bitterness of affliction. Nay, whether God come to His children with a rod or a crown, if He come Himself with it, it is well. Welcome, welcome, Jesus, whatsoever way Thou come, if we can get a sight of Thee! and sure I am it is better to be sick, providing Christ come to the bedside and draw by the curtains and say, "Courage, I am thy salvation," than to enjoy health, being lusty and strong, and never to be visited of God.—*Rutherford.*

